# Diagnostic performance of circulating microRNA signatures for differentiating tuberculosis disease from tuberculosis infection

**DOI:** 10.1007/s00430-025-00853-z

**Published:** 2025-09-24

**Authors:** Anne Ahrens Østergaard, Stephanie Bjerrum, Kristian Assing, Maria Bisgaard Borup, Rasmus Bank Lynggaard, Christiane Abildgaard, Ingrid Louise Titlestad, Torben Tranborg Jensen, Hans Johan Niklas Lorentsson, Ole Hilberg, Christian Morberg Wejse, Søren Feddersen, Isik Somuncu Johansen

**Affiliations:** 1 Research Unit of Infectious Diseases, Department of Clinical Research, University of Southern Denmark, J.B. Winsløws Vej 4, Indgang 20, 5000 Odense C, Denmark; 2 Department of Infectious Diseases, Odense University Hospital, J.B. Winsløws Vej 4, Indgang 20, 5000 Odense C, Denmark; 3 Department of Infectious Diseases, University Hospital of Copenhagen, Opgang 86, 5. Sal, Esther Møllers Vej 6, 2100 RigshospitaletCopenhagen, Denmark; 4 Department of Clinical Immunology, Odense University Hospital, J.B. Winsløws Vej 4, Indgang 5, 5000 Odense C, Denmark; 5 Research Unit of Clinical Immunology, Department of Clinical Research, University of Southern Denmark, J.B. Winsløws Vej 4, Indgang 5, 5000 Odense C, Denmark; 6 Department of Respiratory Medicine, Odense University Hospital, J.B. Winsløws Vej 4, Indgang 20, Odense, Denmark; 7 Department of Clinical Biochemistry, Odense University Hospital, Kløvervænget 47, 5000 Odense C, Denmark; 8 Odense Respiratory Research Unit (ODIN), Department of Clinical Research, University of Southern Denmark, Kløvervænget 2, Indgang 87-88, 5000 Odense C, Denmark; 9 Department for Pulmonary Diseases, Esbjerg Hospital, Finsensgade 35, Bygning E, Etage 3, 6700 Esbjerg, Denmark; 10 Section of Infectious Diseases, Department of Medicine, Herlev and Gentofte Hospital, University of Copenhagen, Gentofte Hospitalsvej 1, 2900 Hellerup, Denmark; 11 Center for Clinical Metabolic Research, Herlev and Gentofte Hospital, University of Copenhagen, Borgmester Ib Juuls Vej 83, 2730 Herlev, Denmark; 12 International Reference Laboratory of Mycobacteriology, Statens Serum Institut, Artillerivej 5, 2300 Copenhagen, Denmark; 13 Department of Medicine, Vejle Hospital, Hospital Lillebælt, Beriderbakken 4, 7100 Vejle, Denmark; 14 Department of Infectious Diseases, Aarhus University Hospital, Palle Juul-Jensens Blvd. 99, 8200 Aarhus, Denmark; 15 Center for Global Health, Department of Public Health, GloHAU, Aarhus University, Bartholins Allé 2, 8000 Aarhus C, Denmark; 16 Clinical Biochemistry, Department of Clinical Research, University of Southern Denmark, Kløvervænget 47, 5000 Odense C, Denmark

**Keywords:** Tuberculosis, Tuberculosis infection, Diagnostic test of tuberculosis, microRNA, Recursive feature elimination, Random forest model

## Abstract

As regulators of innate and adaptive immunity, microRNAs (miRNAs) could aid in the discrimination between tuberculosis disease (TB) and (latent) TB infection (TBI). We analysed 754 circulating miRNAs in participants diagnosed with TB and TBI using TaqMan™ Advanced miRNA Human A and B cards. MiRNAs were normalized exogenously and endogenously via geometric means of selected reference miRNAs. Expression analysis was used to identify miRNAs that were significantly differentially expressed between individuals with TB and those with TBI. We utilised recursive feature elimination with a Random Forest model to identify the miRNAs most effective at discriminating TB from TBI and subsequently validated the miRNA in another group. 95 persons diagnosed with TB or TBI was divided into a discovery group (n = 36) and a validation group (n = 59). In the discovery group, we identified 495 distinct miRNAs in 36 persons with TB or TBI and by recursive feature elimination identified hsa-miR-148a-3p, hsa-miR-204-5p and hsa-miR-584-5p and created a three-miRNA-diagnostic model. In the validation group, the three-miRNA-diagnostic model had poorer performance. Expression analysis revealed 13 significantly differentially expressed miRNAs, including hsa-miR-148a-3p and hsa-miR-204-5p. Subsequent analysis in a validation group consisting of 59 persons revealed that six of the 14 miRNAs, including hsa-miR-148a-3p, exhibited the same pattern, albeit without statistical significance. Three circulating miRNAs showed potential for differentiating TB from TBI in the discovery cohort, but these differences were less pronounced in the validation cohort.

**Supplementary Information:**

The online version contains supplementary material available at 10.1007/s00430-025-00853-z.

## Introduction

Tuberculosis disease (TB) remains a significant global health challenge as more than 10 million fall ill from TB annually [1]. To enhance the identification of cases, novel biomarkers, less correlated to bacillary load and representative sample accessibility, are required [2, 3]. The WHO defines Tuberculosis infection (TBI) as a state of persistent immune response to stimulation by *Mycobacterium tuberculosis* (*Mtb*) antigens with no evidence of clinically manifest TB disease. This is referred to as “TB infection” as distinct from “TB disease” [4] (formerly referred to as latent tuberculosis infection (LTBI)). This response can be detected by Interferon Gamma Release Assays (IGRA) with a specificity of ≥ 97% [5, 6]. However, IGRA cannot discriminate TBI from TB. The management of TBI is an important component of the TB elimination strategy in low TB incidence countries [7].

The response to TB therapy is typically evaluated based on the eradication of acid-fast bacilli, documented through culture or microscopy. This form of verification is mostly applicable to pulmonary (PTB). In persons where the TB diagnosis is not microbiologically confirmed, monitoring treatment response is limited to clinical and possibly radiological assessment [8]. Thus, biomarkers for evaluating TB treatment responses are required, yet the poor associations between previous biomarkers and bacterial load and their use in treatment monitoring appears to be a challenge. Biomarkers released during infection have the potential to be utilised as a tool for monitoring therapy effectiveness [9, 10].

MiRNAs are small (18- to 25-nucleotides), single-stranded, non-coding RNA molecules that regulate gene expression by causing translation blockage or messenger RNA cleavage [11]. Although miRNAs exert their functions intracellularly, they are continuously released from cells and they have a high stability in plasma [12]. The potential of circulating microRNAs (miRNAs) as biomarkers for TB disease has been explored in comparison to healthy controls (HC) [13]. Furthermore, selected miRNAs have been studied as discriminators of TB and TBI [14]. Studies by Nzdi et al. and Kathirvel et al. have demonstrated the relevance of miRNAs for TB diagnosis in populations where TB diagnosis is challenging, including children and people with HIV [15, 16]. MiRNAs, with their dynamic and specific expression profiles, may serve as indicators of treatment response, reflecting changes in bacterial burden and immune modulation during therapy. Nonetheless, the utilisation of miRNAs as biomarkers presents challenges due to the lack of uniform reference values caused by different assay platforms and various normalization methods [17]. In order to establish more uniform normalizations, the geometric means of multiple reference genes have been employed to normalise miRNAs [18, 19], rather than obtaining normalization through reference to a single “housekeeping” miRNA [20].

In this exploratory study, we aimed to investigate the expression profiles of miRNA in persons with TB or TBI at baseline and after initiation of treatment for TB. The objective was to identify candidate miRNAs that could serve as biomarkers to differentiate between TB and TBI, and their ability to monitor treatment response.

## Method

### Patient inclusion

Participants aged over 18 years with a presumptive or laboratory-confirmed diagnosis of TB or TBI were consecutively enrolled upon providing written informed consent at departments of respiratory medicine and infectious diseases across six Danish hospitals (Odense, Esbjerg, Aarhus, Sønderborg, Hillerød and Gentofte) from September 2020 to September 2023. Participants were enrolled both from the inpatient and outpatient setting where the diagnosis of TB disease was made by treating physicians at study sites.

We classified diagnosis of TB disease as Definite TB: Culture isolation of *Mtb*, a positive specific PCR, or the presence of acid-fast bacilli identified by microscopy; Probable TB: Symptoms, radiological or histological findings compatible with TB; Possible TB: Clinical suspicion and response to antituberculous treatment. For our analysis, patients with both PTB and extrapulmonary TB (EPTB) were classified as having PTB. A follow-up evaluation was conducted 8-weeks after initial diagnosis to identify those who were later diagnosed with a different condition or discontinued treatment due to a re-evaluation of their TB status. Patients whose diagnosis was refuted at follow-up were excluded from the final analysis set.

TBI was defined as a positive IGRA, either as part of post-exposure investigation or screening prior to immunosuppressive treatment combined with the absence of TB disease as evaluated by the treating physician. HIV infection was not an exclusion criterion. A follow-up evaluation was conducted two years after inclusion to identify any subsequent diagnosis of TB disease.

### Sample collection

**Fig. 1:**
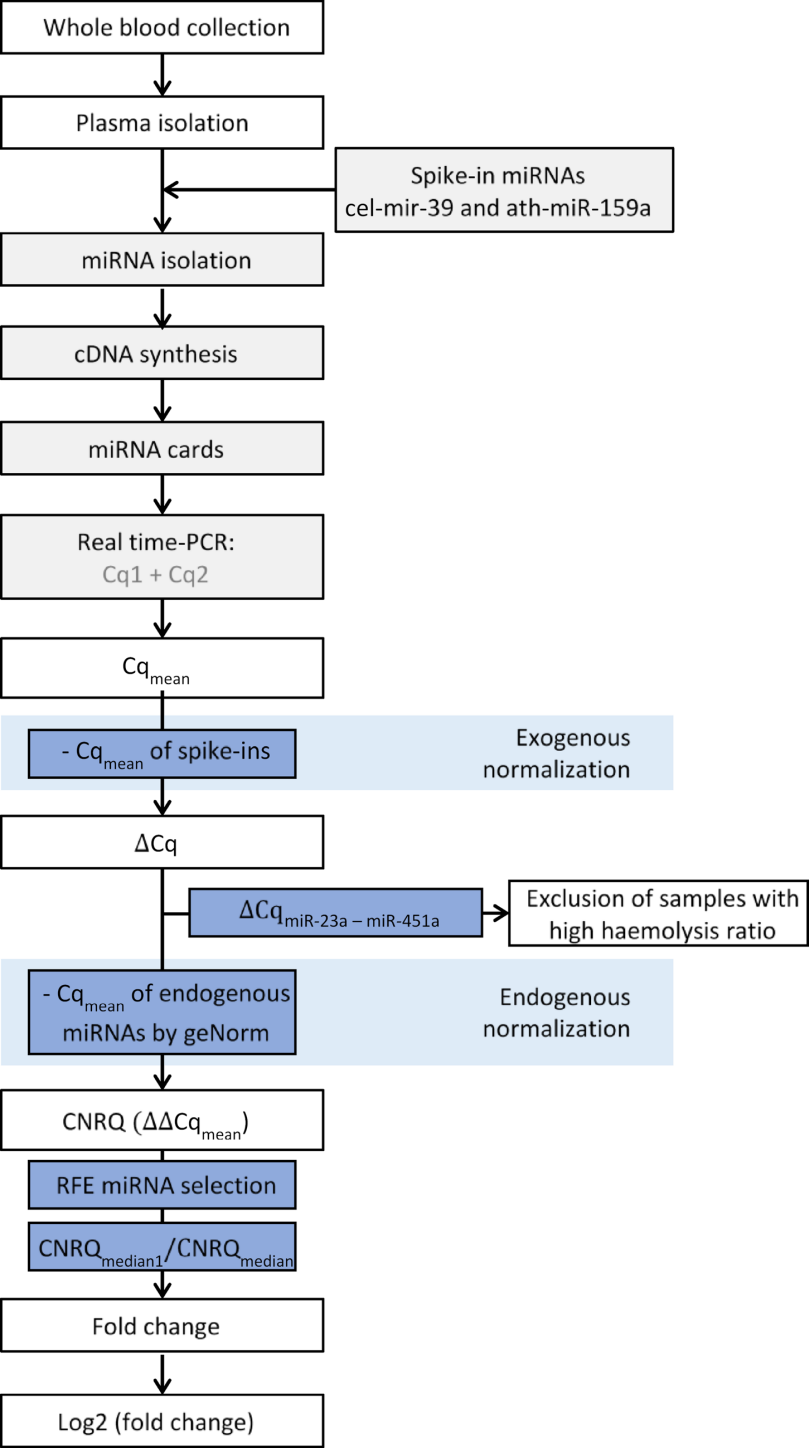
Method flow. *MiRNA* microRNA, *cDNA* complementary deoxyribonucleic acid, *Cq* quantification cycle, *CNRQ* calibrated normalised relative quantities, *log2* the binary logarithm and *PCR* polymerase chain reaction

Peripheral blood was collected in 10 mL K2-EDTA tubes, drawn between 7 am and 3 pm. Within 1 h of collection, plasma was isolated by centrifugation at 2000 × g for 10 min at 20 °C and transferred to RNase-free tubes within 2 h of collection and stored at -70 °C followed by RNA isolation (Fig. 1). For participants with TB, blood was drawn before the initiation or after one dose of antituberculous treatment (baseline) and after 8-weeks of antituberculous treatment (follow-up). For participants with TBI, blood was drawn before the start of any antituberculous treatment.

None of the plasma samples had visible haemolysis. Total RNA, including small RNA, was extracted from 95 (discovery group, n = 36, validation group, n = 59) plasma samples (200 µL of plasma/sample) using the Norgen Total RNA Purification Kit (Norgen Biotek Corp., Thorold, Ontario, Canada) according to the manufacturer’s instructions. RNA was eluted in 50 µL of Elution Solution. For technical normalization, each sample was spiked with 5 fmol synthetic *Arabidopsis thaliana* miR-159a (ath-miR-159a) and *Caenorhabditis elegans* miR-39a (cel-miR-39a).

### Plasma miRNA profiling

The discovery group was intentionally limited in size, based on the hypothesis that TB-specific miRNA signatures would be detectable in a small cohort if truly disease-associated*.* We deliberately selected 18 individuals with TBI and 18 individuals with TB disease (comprising 9 with definite TB and 9 with probable/possible TB) resulting in a total of 50 samples for miRNA profiling. This included 38 baseline samples (TBI n = 18, PTB n = 10, EPTB n = 8), and 12 follow-up samples (TBI n = 8, PTB n = 1 and EPTB n = 3).

We analysed differential miRNA expression across several comparisons: TBI at baseline vs. after 8-weeks without treatment; TB vs. TBI at baseline; TB at baseline vs. after 8-weeks of treatment; PTB vs. EPTB at baseline; and definite vs. probable/possible TB. We did not make any changes to the discovery groups after the initial analyses, to avoid bias or overfitting. We quantified a total of 754 unique miRNAs by semi-quantitative real-time PCR (qRT-PCR) using the TaqMan™ Advanced miRNA Human A and B cards (Applied Biosystems™ Thermo Fisher Scientific Inc.). Total RNA was first converted to cDNA using the TaqMan™ Advanced miRNA cDNA Synthesis Kit (Applied Biosystems™ Thermo Fisher Scientific Inc.) with universal reverse transcription (RT) primers. The mature miRNAs were modified by adding a poly (A) tail (3’) and an adaptor (5’) and the modified miRNAs were then converted to cDNA in a single RT reaction followed by amplification. Both cDNA synthesis and amplification were performed according to the manufacturers’ instructions. Loaded TaqMan™ Advanced miRNA Human A and B cards were cycled in a ViiA7 (Thermo Fisher Scientific Inc.). Data analysis was performed using the QuantStudio Real-Time PCR Software (v.1.3) and the relative threshold algorithm. Cq-values were exported to the qBase^PLUS^ software (Biogazelle, NV, Belgium) for relative quantification.

### Validation of selected miRNAs

In the validation group, we obtained plasma samples from 59 participants; 22 participants with TBI and 37 with TB. We selected 39 miRNAs for further quantification. Based on the observed expression patterns, number of differentially expressed miRNAs, and statistical significance in each comparison, we selected key contrasts for validation: TB vs. TBI, definite vs. probable/possible TB, and TB at baseline vs. follow-up. As described for miRNA profiling, total RNA from these samples was converted to cDNA using the TaqMan™ Advanced miRNA cDNA Synthesis Kit (Applied Biosystems™ Thermo Fisher Scientific Inc.). For each RNA sample, RT was performed in triplicates. Following amplification qPCR (20 µL reactions) was carried out using the StepOnePlus real-time instrument (Applied Biosystems™ Thermo Fisher Scientific Inc.) and each sample was run in singlicate. Thermal cycling conditions were: 95 °C for 20 s followed by 40 cycles of 95 °C for 1 s and 60 °C for 20 s. The ExpressionSuite Software (v1.3) was used to obtain Cq-values, which were exported to the qBase^PLUS^ software (Biogazelle, NV, Belgium) for relative quantification.

### MiRNA normalization and control for haemolysis

We normalised cards A and B separately since one quantification cycle (Cq) difference is expected between card A and B, as card A contain more abundant miRNAs.

The data normalization involved exogenous miRNA as described by Mitchell et al. [21], followed by endogenous normalization. We normalised to the exogenous/spike-in miRNAs duplicate determined cel-miR-39 and ath-miR-159a by

$$ \begin{aligned} \Delta {\text{Cq}} & = {\text{Cqmean}} - \left( {\left( {\frac{{{\text{cel - miR}} - 39_{1} + {\text{cel - miR}} - 39_{2} }}{2}} \right)} \right. \\ & \quad + \left( {\frac{{{\text{ath - miR}} - 159{\text{a}}_{1} + {\text{ ath - miR}} - 159{\text{a}}_{2} }}{2}} \right) \\ & \quad \left. { - \left( {{\text{cel - miR}}39_{{{\text{median}}}} + {\text{ ath - miR}} - 159{\text{a}}_{{{\text{median}}}} } \right)} \right) \\ \end{aligned} $$

After exogenous normalization, we calculated the haemolysis ratio based on haemolysis dependent hsa-miR-451a and haemolysis independent hsa-miR-23a-3p by

$$ \Delta {\text{Cq}}_{{{\text{miR - 23a - 3p {-} miR - 451a}}}} = {\text{ miR - 23a - 3p }}{-}{\text{ miR - 451a}} $$

to exclude samples with haemolysis ratio > 14 [22]. No samples of card A or B had $\Delta$ Cq_miR-23a-3p—miR-451a_ outside the range of −2.2 to 6.1, so no samples were excluded.

The stability of the identified miRNAs was tested using the geNorm function of qBase^PLUS^ (Biogazelle/CellCarta) to calculate M-values for endogenous normalization [18]. We normalised to hsa-miR-142-3p and hsa-miR-191-5p for card A and hsa-miR-361-3p and hsa-miR-425-3p for card B [23]. A fold change was calculated as the ratio of median calibrated normalised relative quantities (CNRQ) between groups.

In the validation group, normalization was performed exogenously and endogenously as described for the cards.

### Statistics and definitions

We assessed normality using QQ plots and histograms. Paired non-parametric miRNA data were compared by Wilcoxon signed-rank test while non-paired non-parametric miRNA data were analysed by median tests, with a *p* < 0.05 considered significant.

We used Stata 18.0/BE (StataCorp LLC, Texas, USA), Scikit-learn [24] and Matplotlib [25] for analyses and graphics. We have utilised k-nearest neighbours’ imputation (based on disease group, biomarker, sex and continuous age) for each disease group in the discovery part to handle missing miRNA values if a miRNA had less than 50% missing values and maximum of 80% missing values per participant. We categorised sex based on the sex specific 10-digit personal identifier (CPR number), which is assigned to all residents at birth or after residing legally in Denmark for three months. We applied recursive feature elimination using a Random Forest model to identify the most potent markers. Recursive feature elimination is a variable selection method that removes the weakest features to enhance model`s predictive accuracy while considering the correlation among variables [26]. We calculated p-values by median test for non-parametrically distributed miRNAs and by Wilcoxon signed-rank test for paired non-parametrically distributed miRNAs. *P*-values below 0.05 were considered significant. We calculated bootstrapped 95% confidence interval (CI)s for values different from 1 and exact binominal 95%CIs for values equal to 1. We chose cut-offs for all miRNAs according to the criteria of minimum of 98% specificity by tabulating sensitivity against specificity to discriminate between TB and TBI for each recursive feature elimination-selected miRNA. We defined the index test as positive for TB disease if all included terms were fulfilled and negative for TB if only part of the terms were fulfilled.

We categorised the TB incidence rate (IR) in the country of birth as follows: low (< 10 TB cases/100,000 population), medium (10–40/100,000), and high (> 40/100,000), based on estimates from the 2019 WHO Global Tuberculosis Report, which was current at the time the study was initiated [27]. For consistency with the study design and patient inclusion period, we used the 2019 incidence estimates originally published by WHO.

### Ethics

The study was conducted in accordance with the Helsinki Declaration and was approved by the Danish Data Protection Agency (Jr: 18/42213 and Jr: 20/45850) and the Danish National Committee of Health Research Ethics (S-20180093).

## Results

### Participant characteristics

**Fig. 2:**
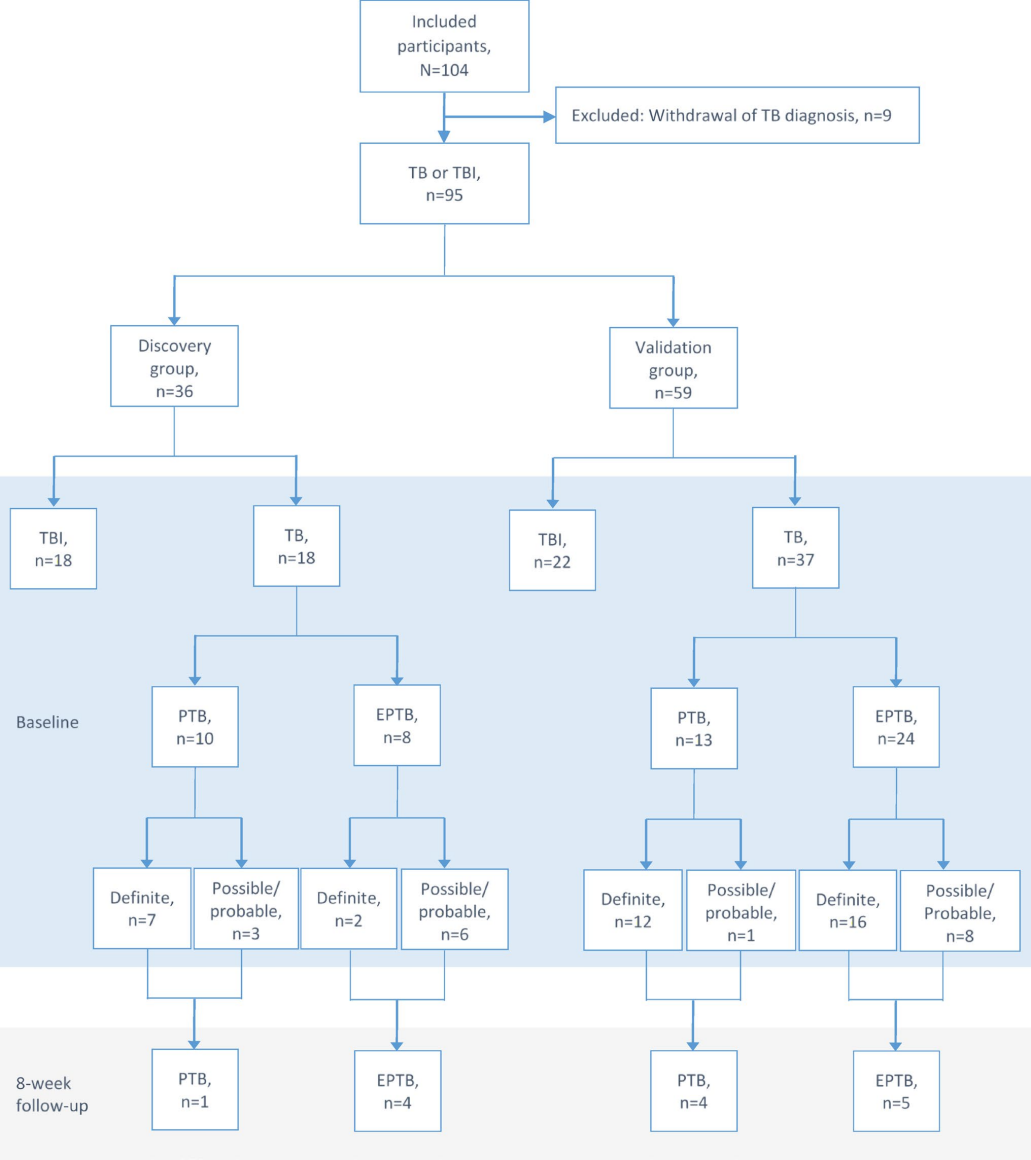
Study participants with tuberculosis infection and tuberculosis disease in discovery and validation group. *EPTB* extrapulmonary tuberculosis, *TB* tuberculosis. *TBI* tuberculosis infection and *PTB* pulmonary tuberculosis. The number and disease site of participants who completed the 8-week follow-up are shown in the 8 week follow-up

Of 104 eligible participants, nine were excluded due to systemic diseases that initially mimicked TB but were later diagnosed as non-TB conditions (diagnoses listed in the Supplementary material). A total of 95 participants were included in the study; 36 in the discovery group, and 59 in the validation group (Fig. 2 and Table 1). The discovery group consisted of 18 participants with TBI and 18 with TB disease, of whom 10 had PTB (55.6%) and 9 were classified as having definite TB (50.0%). The validation group included 22 participants with TBI and 37 with TB, of whom 13 had PTB (35.1%) and 28 were classified as definite TB (75.7%). During the two-year follow-up period, none of the participants with TBI progressed to TB disease.

**Table 1:** Characteristics of discovery and validation groups of participants with tuberculosis and tuberculosis infection

Characteristics	Discovery group			Validation group		
	TBI	TB	Group total	TBI	TB	Group total
n (%)	18 (50.0)	18 (50.0)	36 (100.0)	22 (37.3)	37 (62.7)	59 (100.0)
Median age in years [IQR]	44.3 [35.2–54.5]	48.1 [38.4–60.5]	46.9 [36.5–55.5]	42.7 [34.2–56.2]	38.7 [28.3–51.4]	39.8 [30.3–55.1]
Female, n (%)	12 (66.7)	9 (50.0)	21 (58.3)	13 (59.1)	17 (45.9)	30 (50.8)
Birthplace in Denmark, n (%)	10 (58.8)	4 (22.2)	14 (40.0)	15 (68.2)	10 (27.0)	25 (42.4)
TB incidence in birth country*, n (%)						
Low	10 (58.8)	4 (22.2)	14 (40.0)	16 (72.7)	10 (27.0)	26 (44.1)
Medium	1 ( 5.9)	0 ( 0.0)	1 ( 2.9)	2 ( 9.1)	5 (13.5)	7 (11.9)
High	6 (35.3)	14 (77.8)	20 (57.1)	4 (18.2)	22 (59.5)	26 (44.1)
*IGRA result, n (%)*						
Negative	NA	1 ( 5.6)	1 ( 2.8)	NA	6 (16.2)	6 (10.2)
Positive	18 (100.0)	14 (77.8)	32 (88.9)	22 (100.0)	23 (62.2)	45 (76.3)
Unknown	NA	3 (16.7)	3 ( 8.3)	NA	8 (21.6)	8 (13.6)
*TB diagnosis, n (%)*						
Definite	NA	9 (50.0)	9 (25.0)	NA	28 (75.7)	28 (47.5)
Probable	NA	8 (44.4)	8 (22.2)	NA	7 (18.9)	7 (11.9)
Possible	NA	1 ( 5.6)	1 ( 2.8)	NA	2 ( 5.4)	2 ( 3.4)
*TB site, n (%)*						
PTB	NA	10 (55.6)	10 (27.8)	NA	13 (35.1)	13 (22.0)
EPTB	NA	8 (44.4)	8 (22.2)	NA	24 (64.9)	24 (40.7)

*EPTB* extrapulmonary tuberculosis disease, *IQR* Interquartile range, *NA* Not applicable, *PTB* pulmonary tuberculosis disease, *TB* tuberculosis disease and *TBI* tuberculosis infection. *TB incidence rate in birth country according to the World Health Organization, 2019

The median age (IQR) was 44.3 years (35.2–54.5) for TBI participants and 48.1 (38.4–60.5) for TB patients in the discovery group. The median age was 42.7 years (34.2–56.2) for TBI participants and 38.7 (28.3–51.4) for patients with TB disease in the validation group. Females constituted 58.3% in the discovery group and 50.8% in the validation group. The minority of the discovery group (40.0%) and validation group (38.5%) were born in Denmark. More TB patients were born in a high TB incidence country, applicable for 14/18 (77.8%) TB patients in the discovery group and 22/37 (59.5%) TB patients in the validation group. Of the patients with TB disease in the discovery group, 14/18 (77.8%) and 23/37 (62.2%) in the validation group had positive IGRA. One patient in the discovery group with EPTB and two patients within the validation group (EPTB, n = 1 and PTB, n = 1) had type II diabetes mellitus. In the validation group, one participant with TBI and two with PTB were living with HIV. The supplement includes participant characteristics in discovery and validation subgroups (Table S1) and a more detailed list of comorbidities.

### Circulating miRNA expression for differentiation between TB and TBI

No samples were excluded due to haemolysis. Among the 754 exogenously and endogenously normalised miRNAs, we detected 495 different miRNAs in the discovery dataset, including those used for normalization. Prior to spike-in normalization, no significant difference was observed in concentration of synthetic miRNAs (Cq_mean_ cel-miR-39 and ath-miR-159a) between TB and TBI. Among 491 miRNAs, 13 were significantly different in the median CNRQ between TB and TBI (Table 2**)**. Recursive feature elimination designated hsa-miR-148a-3p, hsa-miR-204-5p and hsa-miR-584-5p as the most important miRNAs for differentiating TB from TBI (Supplementary Figure S1). Supplementary Figure S2 shows a volcano plot of exogenously and endogenously normalised miRNAs in the discovery group and Supplementary Figure S3 shows a volcano plot of spike-in normalised $\Delta$ Cqs.

**Table 2:** MiRNA levels of recursive feature elimination selected miRNAs in discovery and validation group

Group	Significantly different miRNAs	RFE selected miRNAs	Sequence	Discovery		Validation	
				Fold change^1^	*p*-value^2^	Fold change	*p*-value^2^
TB vs TBI	hsa-let-7a-5p		UGAGGUAGUAGGUUGUAUAGUU	0.950	0.046	0.913	0.086
	hsa-let-7e-3p		CUAUACGGCCUCCUAGCUUUCC	1.15	0.046	0.918	0.465
	hsa-let-7f-5p		UGAGGUAGUAGAUUGUAUAGUU	0.918	0.046	0.949	0.086
	hsa-miR-1301-3p		UUGCAGCUGCCUGGGAGUGACUUC	0.949	0.046	0.954	0.144
	hsa-miR-139-5p		UCUACAGUGCACGUGUCUCCAGU	0.860	0.046	1.02	0.661
	hsa-miR-146a-5p		UGAGAACUGAAUUCCAUGGGUU	0.944	0.046	0.999	0.920
	hsa-miR-148a-3p	hsa-miR-148a-3p	UCAGUGCACUACAGAACUUUGU	1.18	0.008	1.12	0.329
	hsa-miR-188-5p		CAUCCCUUGCAUGGUGGAGGG	1.20	0.046	1.14	0.465
	hsa-miR-190a-5p		UGAUAUGUUUGAUAUAUUAGGU	0.824	0.046	1.043	0.661
	hsa-miR-204-5p	hsa-miR-204-5p	UUCCCUUUGUCAUCCUAUGCCU	1.57	0.008	1.00	0.920
	hsa-miR-22-5p		AGUUCUUCAGUGGCAAGCUUUA	1.08	0.046	0.992	1.00
	hsa-miR-33a-5p		GUGCAUUGUAGUUGCAUUGCA	1.11	0.046	0.926	0.465
	hsa-miR-374b-5p		AUAUAAUACAACCUGCUAAGUG	0.942	0.046	1.01	0.661
		hsa-miR-584-5p	UUAUGGUUUGCCUGGGACUGAG	0.944	0.505	0.958	0.465
Definite vs probable/possible	hsa-miR-1227-3p		CGUGCCACCCUUUUCCCCAG	0.936	0.046	–	–
	hsa-miR-191-3p	hsa-miR-191-3p	GCUGCGCUUGGAUUUCGUCCCC	0.801	0.001	1.056	0.639
	hsa-miR-221-5p	hsa-miR-221-5p	ACCUGGCAUACAAUGUAGAUUU	1.148	0.018	1.071	0.639
	hsa-miR-24–2-5p	hsa-miR-24–2-5p	UGCCUACUGAGCUGAAACACAG	0.829	0.004	1.01	0.639
	hsa-miR-32-3p	hsa-miR-32-3p	CAAUUUAGUGUGUGUGAUAUUU	1.15	0.001	0.976	0.639
	hsa-miR-330-3p		GCAAAGCACACGGCCUGCAGAGA	0.738	0.018	1.137	0.291
	hsa-miR-339-5p		UCCCUGUCCUCCAGGAGCUCACG	0.870	0.018	0,903	0.044
	hsa-miR-411-3p		UAUGUAACACGGUCCACUAACC	1.12	0.018	1.11	0.160
	hsa-miR-655-3p		AUAAUACAUGGUUAACCUCUUU	1.13	0.018	1.199	0.772
EPTB vs PTB	hsa-miR-1227-3p		CGUGCCACCCUUUUCCCCAG	1.30	0.019	–	–
	hsa-miR-182-5p	hsa-miR-182-5p	UUUGGCAAUGGUAGAACUCACACU	0.639	0.058	1.14	0.109
	hsa-miR-191-3p	hsa-miR-191-3p	GCUGCGCUUGGAUUUCGUCCCC	0.766	0.004	1.05	0.639
	hsa-miR-221-5p		AGCUACAUUGUCUGCUGGGUUUC	0.869	0.004	1.02–	0.639
	hsa-miR-374b-5p		AUAUAAUACAACCUGCUAAGUG	1.08	0.058	0.849	0.362
	hsa-miR-548e-3p	hsa-miR-548e-3p	AAAAACUGAGACUACUUUUGCA	1.26	0.004	1.02	0.639
TB visit 1 vs visit 2	hsa-let-7a-5p		UGAGGUAGUAGGUUGUAUAGUU	1.42	0.043	1.03	0.441
	hsa-let-7f-2-3p		CUAUACAGUCUACUGUCUUUCC	1.41	0.043	0.997	0.465
		hsa-let-7i-5p	UGAGGUAGUAGUUUGUGCUGUU	1.26	0.225	0.918	0.051
		hsa-miR-1256	AGGCAUUGACUUCUCACUAGCU	1.39	0.414	0.96	0.314
	hsa-miR-151b		UCGAGGAGCUCACAGUCU	1.31	0.043	1.35	0.144
	hsa-miR-15b-3p		CGAAUCAUUAUUUGCUGCUCUA	1.08	0.043	–	–
		hsa-miR-181c-3p	AACCAUCGACCGUUGAGUGGAC	1.17	0.138	–	–
	hsa-miR-188-5p		CAUCCCUUGCAUGGUGGAGGG	1.12	0.043	0.952	0.725
	hsa-miR-199a-5p		CCCAGUGUUCAGACUACCUGUUC	1.01	0.043	0.895	0.314
	hsa-miR-200b-3p		UAAUACUGCCUGGUAAUGAUGA	1.03	0.043	–	–
		hsa-miR-221-5p	ACCUGGCAUACAAUGUAGAUUU	1.37	0.686	1.02	0.068
		hsa-miR-23a-3p	AUCACAUUGCCAGGGAUUUCC	1.23	0.138	1.00	0.086
	hsa-miR-424-3p		CAAAACGUGAGGCGCUGCUAU	1.30	0.043	0.929	0.859
	hsa-miR-450a-5p		UUUUGCGAUGUGUUCCUAAUAU	1.10	0.043	–	–
	hsa-miR-548k	hsa-miR-548k	AAAAGUACUUGCGGAUUUUGCU	1.09	0.043	0.971	0.144
	hsa-miR-625-5p		AGGGGGAAAGUUCUAUAGUCC	1.31	0.043	0.977	0.260
		hsa-miR-629-5p	UGGGUUUACGUUGGGAGAACU	0.922	0.043	1.15	0.214
	hsa-miR-7–1-3p		CAACAAAUCACAGUCUGCCAUA	1.34	0.043	–	–

*EPTB* extrapulmonary tuberculosis disease, *PTB* pulmonary tuberculosis disease, *TB* tuberculosis disease, *TBI* tuberculosis infection, *RFE* recursive feature elimination.–: Unavailable

^1^Fold change in calibrated normalised relative quantities (CNRQ)

^2^Calculated by median test for all groups except TB visit 1 vs TB visit 2 calculated by Wilcoxon signed-rank test (paired data)

**Fig. 3:**
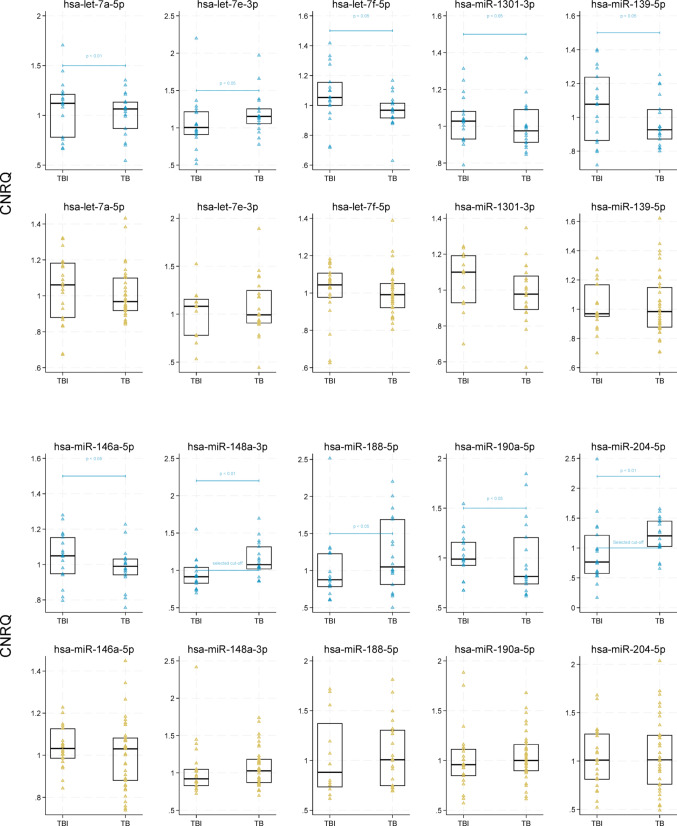
Circulating miRNA expression for differentiation between TB and TBI. Scatterplot of calibrated normalised relative quantities (CNRQ) values of recursive feature elimination selected and significantly different miRNAs in discovery group and the same miRNAs in validation group of TB and TBI. *P*-value (*p*) calculated by median test for discovery group (light blue) and the same miRNAs in validation group (sand) showing only significant findings. Box shows median and quartiles of both groups (black). Selected cut-off for test positive for TB (light blue) selected by recursive feature elimination. *TB* tuberculosis disease, *TBI* tuberculosis infection

**Fig. 4:**
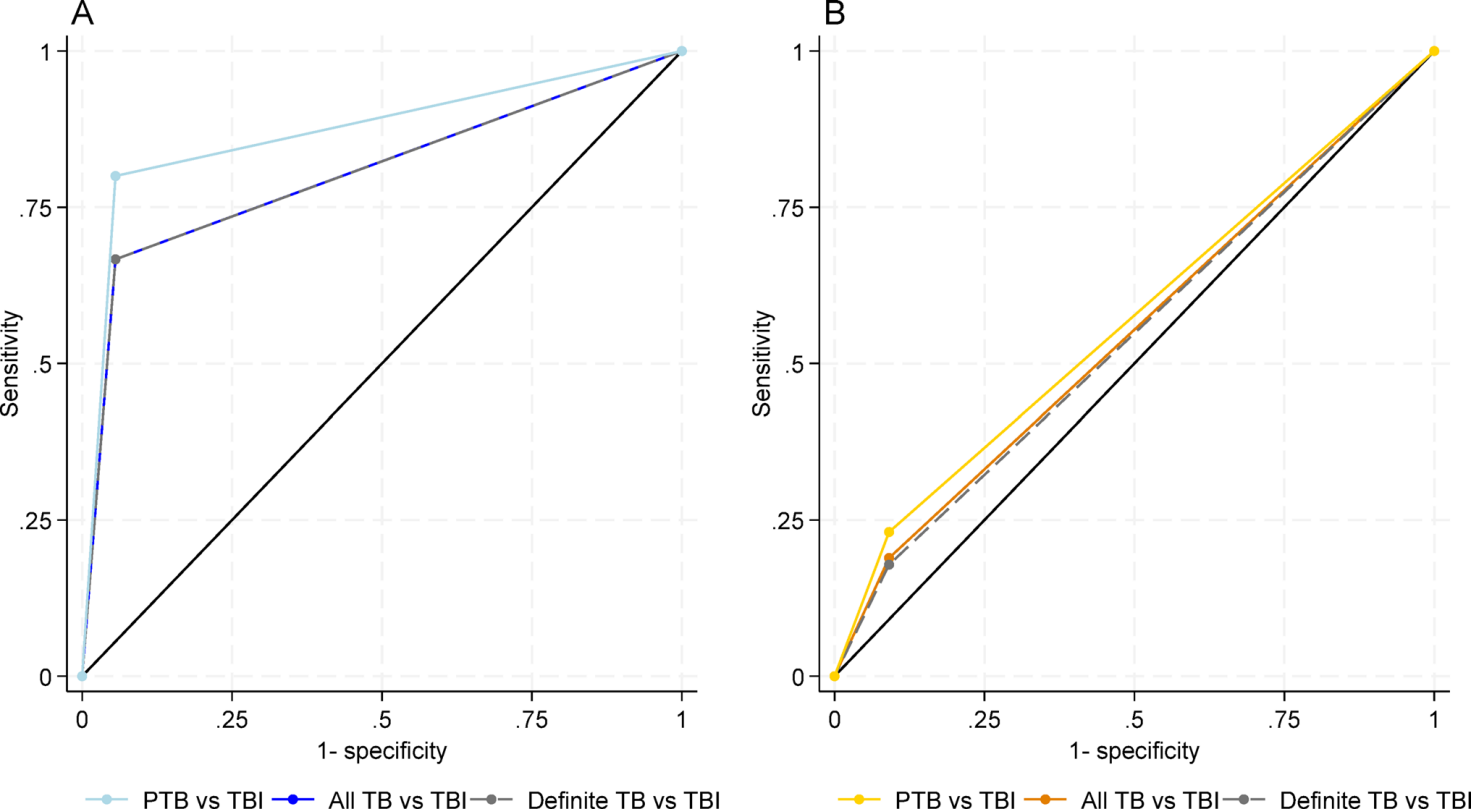
Receiver operating characteristic curves for index test of the three-miRNA-diagnostic model. Hsa-miR-148a-3p, hsa-miR-204-5p and hsa-miR-584-5p differentiation of TB from TBI in discovery group (figure A) and validation group (figure B). **A**. Discovery group: All TB against TBI (light blue): Area under ROC curve = 0.81 (95%CI: 0.69–0.92), n = 36. Definite TB cases against TBI (grey dashed): Area under ROC curve = 0.81 (95%CI: 0.63–0.98), n = 27. PTB against TBI (blue): Area under ROC curve = 0.87 (95%CI: 0.73–1.01), n = 28. **B.** Validation group: All TB against TBI (yellow): Area under ROC curve = 0.55 (95%CI: 0.46–0.64) n = 59. Definite TB cases against TBI (grey dashed): Area under ROC curve = 0.54 (95% CI: 0.44–0.64), n = 50. PTB against TBI (orange): Area under ROC curve = 0.57 (95%CI: 0.44–0.70), n = 35. *PTB* pulmonary tuberculosis disease, *TB* tuberculosis disease, *TBI* tuberculosis infection

The median hsa-miR-148a-3p and hsa-miR-204-5p CNRQ values were upregulated, and the median hsa-miR-584-5p CNRQ was downregulated in TB disease compared to TBI (Fig. 3 light blue). To create an index test for TB disease, we constructed a three-miRNA-diagnostic model, selecting cut-off values to maximise specificity, (Table 3). In the discovery group, the three-miRNA-diagnostic model differentiated TB disease from TBI with a sensitivity of 67% (95%CI: 44–89), specificity of 94% (95%CI: 84–105) and an area under the ROC curve of 0.81 (95%CI: 0.69–0.92) (Fig. 4). A subgroup analysis including only participants with definite TB disease showed minimal variation in circulating miRNA levels compared to the overall TB group.

**Table 3:** MiRNA cut-offs for the three-miRNA-diagnostic model

		Index test miRNAs	Selected cut-off values for index test positive for TB:	Sensitivity (95% CIs)	Specificity (95% CIs)	AUC (95% CIs)	PPV (95% CIs)	NPV (95% CIs)
Discovery group	TB Contra TBI	hsa-miR-148a-3p	≥ 1 CNRQ	67% (44–89)	94% (84–105)	0.81 (0.69–0.92)	0.92 (0.78–1.06)	0.74 (0.55–0.92)
		hsa-miR-204-5p	≥ 1 CNRQ					
		hsa-miR-584-5p	≤ 1.15 CNRQ					
	Subgroup analysis							
	Definite TB contra TBI	hsa-miR-148a-3p	≥ 1 CNRQ	67% (33–100)	94% (84–105)	0.81 (0.63–0.98)	0.86 (0.59–1.1)	0.85 (0.68–1.01)
		hsa-miR-204-5p	≥ 1 CNRQ					
		hsa-miR-584-5p	≤ 1.15 CNRQ					
	PTB contra TBI	hsa-miR-148a-3p	≥ 1 CNRQ	80% (55–105)	94% (83–105)	0.87 (0.73–1.01)	0.89 (0.67–1.10)	0.89 (0.75–1.04)
		hsa-miR-204-5p	≥ 1 CNRQ					
		hsa-miR-584-5p	≤ 1.15 CNRQ					
Validation group	TB contra TBI	hsa-miR-148a-3p	≥ 1 CNRQ	19% (6.7–31)	91% (78–103)	0.55 (0.46–0.64)	0.78 (0.49–106)	0.40 (0.26–0.54)
		hsa-miR-204-5p	≥ 1 CNRQ					
		hsa-miR-584-5p	≤ 1.15 CNRQ					
	Subgroup analysis							
	Definite TB contra TBI	hsa-miR-148a-3p	≥ 1 CNRQ	18% (2.9–33)	91% (78–104)	0.54 (0.44–0.64)	71 (0.34–109)	0.47 (0.31–0.62)
		hsa-miR-204-5p	≥ 1 CNRQ					
		hsa-miR-584-5p	≤ 1.15 CNRQ					
	PTB contra TBI	hsa-miR-148a-3p	≥ 1 CNRQ	23% (0.38–46)	91% (79–103)	0.57 (0.44–0.70)	0.60 (0.13–107)	0.67 (0.48–0.84)
		hsa-miR-204-5p	≥ 1 CNRQ					
		hsa-miR-584-5p	≤ 1.15 CNRQ					

*AUC* Area under curve, *CNRQ* Calibrated normalised relative quantities, *NPV* Negative predictive value, *PPV* Positive predictive value, *TB* Tuberculosis disease, *TBI* Tuberculosis infection

In the validation group, we observed no fold change in hsa-miR-204-5p and hsa-miR-584-5p, both remaining at 1.00. While hsa-miR-148a-3p, hsa-miR-188-5p and hsa-miR-33a-5p showed increased expression in TB patients, they were not significantly different compared to persons with TBI. Similarly, hsa-let-7a-5p, hsa-let7f-5p, and hsa-1301-3p were decreased in persons with TB relative to TBI both in the validation and discovery groups, although not significantly. Hsa-let-7e-3p and hsa-miR-190a-5p showed opposite expressions in the discovery and validation groups.

The three-miRNA-diagnostic model, using the same cut-offs as in the discovery group, differentiated TB from TBI with a sensitivity of 19% (95%CI: 6.7–31), a specificity of 91% (95%CI: 78–103) and an area under the ROC curve of 0.55 (95%CI: 0.46–0.64).

### Subgroup exploration and analysis

#### Circulating miRNA expression for differentiation between definite TB and possible/probable TB

**Fig. 5:**
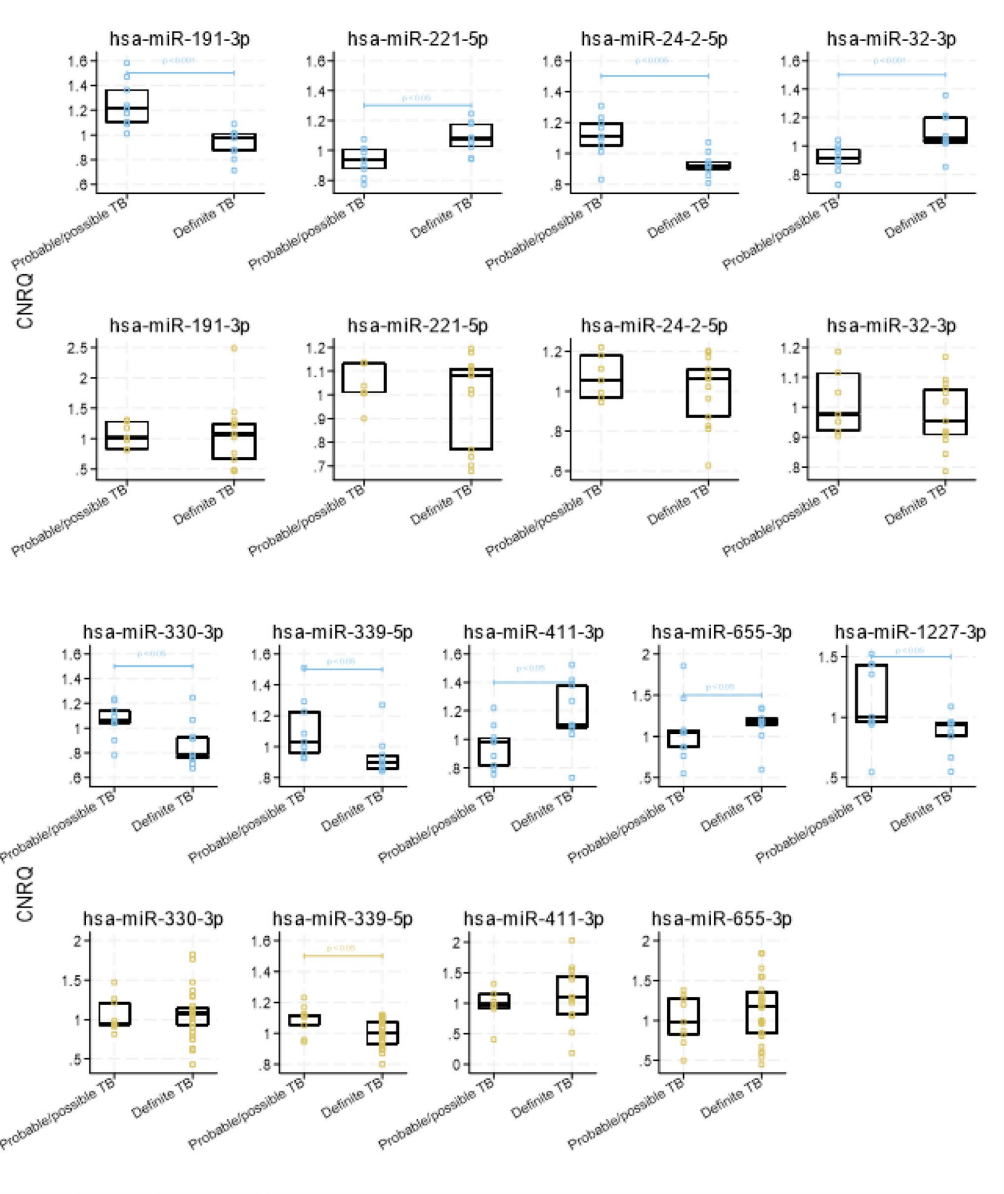
Circulating miRNA expression for differentiation between definite TB and possible/probable TB. Scatterplot of calibrated normalised relative quantities (CNRQ) values of recursive feature elimination selected miRNAs and significant miRNAs in discovery group of definite and probable/possible TB. No data in the validation group for hsa-miR-1227-3p. *TB* tuberculosis disease

Further analysis in the discovery group explored differences in miRNA profiles between cases of definite TB and those classified as probable/possible TB. We identified hsa-miR-191-3p, hsa-miR-221-5p, hsa-miR-24–2-5p and hsa-miR-32-3p as key miRNAs for differentiating definite TB from probable/possible TB using recursive feature elimination. The median CNRQ levels of hsa-miR-191-3p and hsa-miR-24–2-5p were downregulated in definite TB while hsa-miR-221-5p and hsa-miR-32-3p were upregulated (Fig. 5). In the validation group, hsa-miR-339-5p was decreased in definite TB (*p* = 0.044). In the validation group hsa-miR-191-3p and hsa-miR-330-3p were marginally increased in definite TB and hsa-miR-1227-3p could not be determined.

When analysing definite TB versus TBI, the sensitivity, specificity and area under the ROC curve for the three-miRNA-diagnostic model remained consistent, regardless if definite or probable/possible TB cases were included across both the discovery and validation groups (Table 3).

#### Circulating miRNA expression for differentiation between PTB and EPTB

**Fig. 6:**
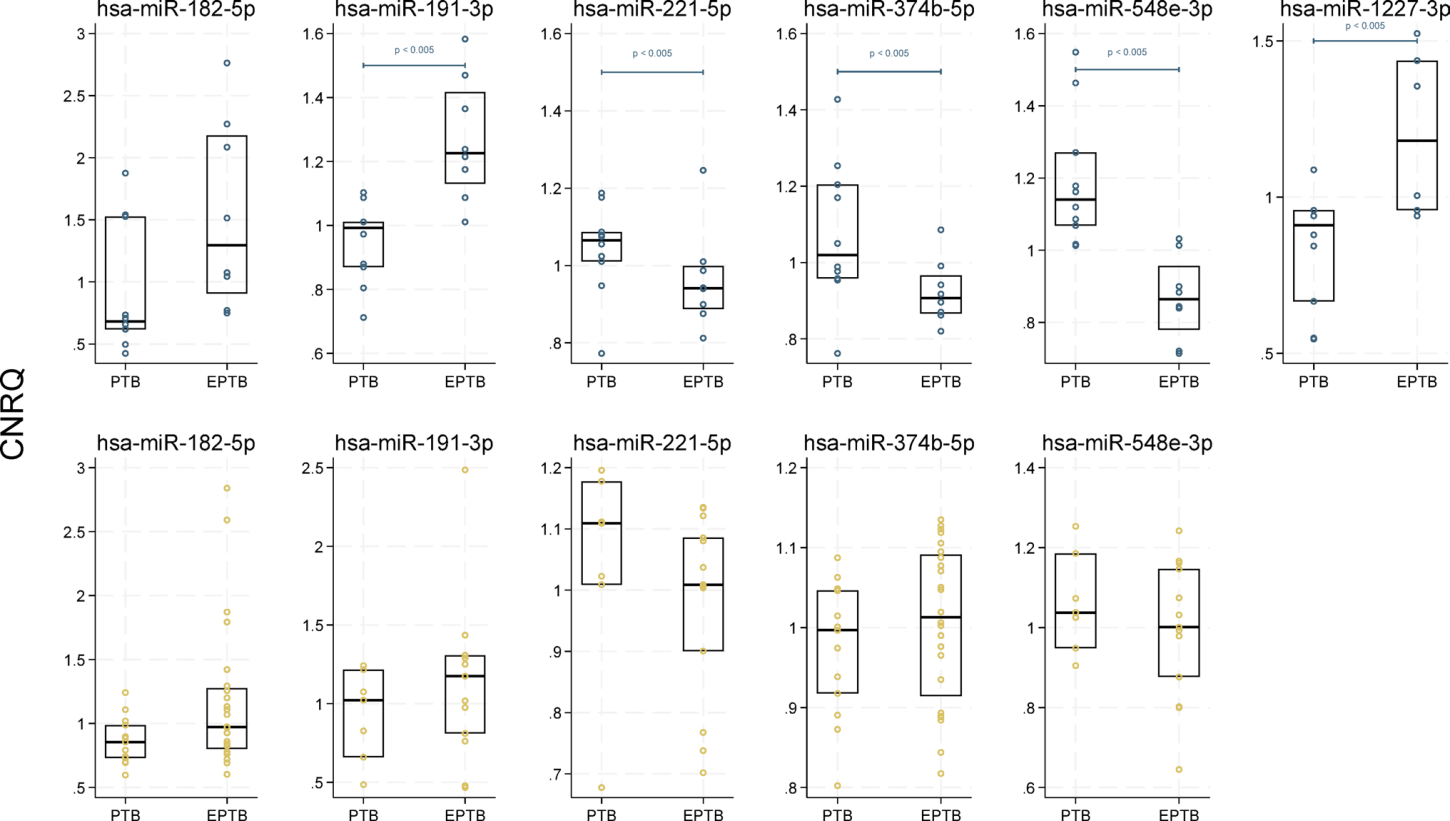
Circulating miRNA expression for differentiation between PTB and EPTB. Scatterplot of calibrated normalised relative quantities (CNRQ) values of recursive feature elimination selected and significant miRNAs in discovery group and validation group of PTB and EPTB. *P*-value (*p*) calculated by median test for discovery group (grey-blue) and validation group (sand) of PTB and EPTB showing only significant findings. Box shows median and quartiles of group (black). Hsa-miR-182-5p, hsa-miR-191-3p, hsa-miR-374b-5p and hsa-miR-548e-3p were selected by recursive feature elimination. No data in the validation group for hsa-miR-1227-3p. *EPTB* extrapulmonary tuberculosis disease, *PTB* pulmonary tuberculosis disease

Comparing miRNA expression profiles of PTB and EPTB, revealed that hsa-miR-182-5p, hsa-miR-191-3p and hsa-miR-548e-3p showed the most significant differential expression between the groups. The median CNRQ values of hsa-miR-191-3p were upregulated, while hsa-miR-548e-3p were downregulated in EPTB compared to PTB (Fig. 6). In the validation group, hsa-miR-182-5p, hsa-miR-191-3p, hsa-miR-221-5p and hsa-miR-548e-3p showed the same pattern as in the discovery group, though these changes were not statistically significant. A subgroup analysis of the index test was performed, with only PTB against TBI. We found a sensitivity of 80% (95%CI: 55–105), a specificity of 94% (95%CI: 83–105) and an area under the ROC curve of 0.87 (95%CI: 0.73–1.01) in the discovery group. In the validation group, the index test to differentiate PTB from TBI demonstrated a sensitivity of 23% (95%CI: 0.38–46), a specificity of 91% (95%CI: 79–103) and an area under the ROC curve of 0.57 (95%CI: 0.44–0.70).

#### Circulating miRNA expression in TB treatment monitoring

Using recursive feature elimination, let-7i-5p, hsa-miR-1256, hsa-miR-181c-3p, hsa-miR-221-5p, hsa-miR-23a-3p, hsa-miR-548k and hsa-miR-629a-5p were identified as key features, all showing downregulation after 8-weeks of treatment (Supplementary Figure S4). We also assessed changes in miRNAs previously identified as distinguishing TBI from TB, specifically hsa-miR-148a-3p, hsa-miR-204-5p and hsa-miR-584-5p, and found no significant differences in their expressions after 8-weeks of treatment (data not shown). In the validation group, hsa-miR-23, hsa-miR-424-3p, hsa-miR-199a-5p, hsa-miR-629-5p and hsa-let-7a-5p exhibited a similar downregulation pattern as observed in the discovery group, though the results were not statistically significant. Baseline and after 8-weeks of treatment levels of hsa-miR-15b-3p, hsa-miR-181c-3p, hsa-miR-200b-3p, hsa-miR-450a-5p and hsa-miR-7–1-3p could not be determined in the validation group.

## Discussion

In this study, we explored the expression of 754 circulating miRNAs in participants with TBI and TB disease to identify potential miRNAs as add-on non-invasive diagnostic biomarkers and created a three-miRNA-diagnostic model. In TB patients, we found that hsa-miR-148a-3p and hsa-miR-204-5p were upregulated, and hsa-miR-584-5p was downregulated, discriminating TB from TBI with an area under the ROC curve of 0.81. The area under the ROC curve was unchanged in the subgroup analysis focusing only on definite TB compared to TBI. Conversely, the analysis of PTB against TBI increased the area under the ROC curve to 0.87. Yet, the expression of hsa-148a-3p, hsa-miR-204-5p and hsa-miR-584-5p did not differ significantly between TB and TBI in the validation group. In the validation group however, hsa-miR-148a-3p and hsa-584-5p showed the same direction of expression as did five of the 14 miRNAs originally identified in the discovery group as being significantly differentially expressed between TB and TBI patients. The three-miRNA-diagnostic model had poorer performance in the validation group as a consequence of smaller differences in miRNA expression.

Several studies have attempted to demonstrate the ability of circulating miRNAs as potential biomarkers in TB diagnosis and TBI screening [13, 14, 28–30]. However, candidate signature miRNAs have not yet been identified for routine diagnostics in TB. In a meta-analysis, miR-29, miR-31, miR-125b, miR-146a and miR-155 were consistently reported for TB diagnosis with an overall sensitivity and specificity of 87.9% (81.7–92.2) and 81.2% (74.5–86.5), respectively [31]. Of note, the studies in the meta-analysis were highly heterogeneous in terms of study population, sample type (serum, plasma, whole blood, peripheral blood mononuclear cells, sputum and urine), grouping miRNAs from ‘3 and ‘5 end as one, method of normalization, discovery and validation. Following validation none of these miRNAs were found to be significantly differentially expressed between TBI and TB patients in our study.

The miRNAs included in the three-miRNA-diagnostic model have been described previously; Hsa-miR-148a-3p has been shown to repress inflammatory gene expression [17, 32] and was found to be increased in plasma from individuals with PTB compared to HC, with normalization of endogenous levels by an index of Hsa-let-7d/g/7i [33]. We found hsa-miR-148a-3p to be upregulated in TB disease compared to TBI in both the discovery and validation group, though this was not statistically significant in the validation group.

Hsa-miR-204-5p is downregulated in cancers and has been hypothesized to promote apoptosis for bacterial clearance [30, 32, 34]. We found hsa-miR-204-5p to be upregulated in patients with TB in the discovery group only.

Hsa-miR-584-5p has been reported to be upregulated in pulmonary non-tuberculous mycobacteria (NTM), distinguishing it from HC with sensitivity of 75.0%, specificity of 66.7% and area under the ROC curve of 0.71 [35]. It is also described as a tumor suppressor in non-small cell lung cancer [36] and found to be downregulated in sepsis [37]. In contrast, we observed hsa-miR-584-5p to be downregulated in TB compared to TBI.

Chakrabarty et al. reported upregulation of hsa-miR-146a-5p in serum from individuals with PTB compared to HC [38]. In contrast, we found downregulation of hsa-miR-146a-5p in TB disease compared to TBI, consistent with Kathirvel et al. in children with PTB [16] and by Tu et al., who reported reduced levels in PTB compared to HC [39]. Hsa-miR-146a-5p modulate by suppressing pro-inflammatory cytokine secretion through TLR4 downregulation [40], however, it is also involved in a broad range of inflammatory conditions [41, 42], indicating its role is not specific to TB.

We found hsa-miR-148a-3p to be upregulated and hsa-miR-584-5p to be downregulated in TB disease compared to TBI, and we hypothesise that, in general, the selected miRNAs represent (pulmonary) inflammation rather than TB-specific immune regulation.

Hsa-let-7a-5p, which regulates innate immune response and is reduced in urosepsis [43, 44], was downregulated in TB disease in both our discovery and validation groups. Duffy et al., found lower levels in household contacts progressing to TB [45]. De Araujo et al. also reported hsa-let-7a-5p as part of predictive model distinguishing TB disease from TBI with an area under the ROC of 0.86 and found its expression associated with *Mtb*-stimulated macrophages and peripheral blood mononuclear cells [46].

Hsa-let-7f-5p has been reported to be downregulated in sepsis and further reduced in septic shock [37]. Similarly, we observed downregulation in TB disease compared to TBI, suggesting a role in inflammation.

Hsa-miR-411-3p and hsa-miR-221-5p were previously found to be downregulated in PTB compared to HC [39] and both have been described as tumour suppressors [47, 48]. In contrast, we found hsa-miR-221-5p upregulated in PTB and definite TB compared to EPTB and probable/possible TB in both the discovery and validation group. Hsa-miR-411-3p was also upregulated in definite TB compared to probable/possible TB in both the discovery and validation group. Çavuşoğlu et al. assessed 83 miRNAs in TB, TBI and HC and reported no differential expression between groups or over the course of treatment [14].

Only a few studies have compared miRNA expression between PTB and EPTB. While Liang et al. and Spinelli et al. identified site-specific miRNA signatures in spinal and pleural TB, respectively [49, 50], we were unable to identify distinct miRNA patterns that reliably differentiate PTB from EPTB in our cohort.

Overall, key challenges in miRNAs research in TB include the lack of standardized normalization methods, the clinical heterogeneity of TB, and frequent comparisons to HC, which risk capturing non-specific inflammation rather than TB-specific signatures. However, X-ray findings were not included in the dataset, and the number of patients with PTB in the study (n = 26) is too small to support meaningful correlation analyses of pulmonary inflammation. MiRNAs profiling is further complicated by platform dependent variability, as demonstrated by Hong et al. [51], who recommend validation using qPCR to ensure accuracy. In this study, we explored a very broad panel of 754 miRNAs rather than pre-selecting candidates from the literature, and validated key findings in an independent patient cohort using qPCR. To address analytical variability, we applied both exogenous and endogenous normalization [17, 18]. We implemented exogenous normalization using two synthetic miRNAs and observed no differences between TB and TBI in the spike-ins, contrasting with previous findings [52]. GeNorm was used for identification of reference miRNAs in the discovery group. Four different miRNAs were selected for endogenously normalization of the cards; card A and B were each normalized with two miRNAs. The same miRNAs were used for endogenously normalization of the validation group, when validating miRNAs as recommended [19].

Minor differences in age and sex distribution across all groups were noted, potentially introducing bias. However, we included both variables in the imputation model for missing data to mitigate this effect. We analysed both significantly expressed miRNAs and those selected through recursive feature elimination to optimise data interpretation [53]. While we included PTB, EPTB and probable/possible TB rather than only definite PTB, subgroup analysis showed minimal variation in miRNA levels among definite TB cases, suggesting limited influenced of bacillary load. The present study has grouped PTB and EPTB into one TB disease group. Though not directly compared in the study by Chakrabarty et al., smaller changes in hsa-mir-146–1, miR-125b and MTB-mir-5 have been described, thereby providing support for our findings of smaller changes in the expression of microRNAs between PTB and EPTB [38]. However, a higher proportion of EPTB compared to PTB in the validation group may have reduced the performance of the three-miRNA model in the validation cohort.

The country of origin differed between the discovery and validation groups, which may have introduced a bias due to variations in host genetics or differing *Mtb* strain lineages. Additionally, the arbitrary (non-random) selection of the discovery group may have contributed to the lack of validation.

In recent years, there has been a paradigm shift in understanding TB, from a binary TB disease/TBI model to a more nuanced spectrum [54]. This study used the older classification, which may have been too broad and unevenly distributed, potentially contributing to the lack of validation. Additional limitations include the small sample size, single time-point miRNA measurement in the discovery group, absence of non-TB inflammatory controls, and retrospective selection of cut-off points, which may have led to overestimation of diagnostic accuracy.

## Conclusion

Circulating miRNAs were explored as a blood-based add-on biomarkers to differentiate TB from TBI, including PTB, EPTB and TB without microbiological confirmation. Several miRNAs with the potential to differentiate TB from TBI were identified in the discovery group but these findings could not be confirmed in the validation group. However, the expression-pattern for some miRNAs in the validation group were similar to those in the discovery group.

## Supplementary Information

Below is the link to the electronic supplementary material.

Supplementary file1 (DOCX 568 kb)

## Data Availability

The datasets generated and/or analyzed during the current study are not publicly available due to the General Data Protection Regulation by the European Data Protection Regulation. Still, they are available from the corresponding author on reasonable request.
