# Supplementary material for: Diagnostic performance of circulating microRNA signatures for differentiating tuberculosis disease from tuberculosis infection
Source: Med Microbiol Immunol. 2025 Sep 24;214(1):45. doi: 10.1007/s00430-025-00853-z (PMC12460509; doi:10.1007/s00430-025-00853-z)
Supplement: Supplementary file 1 — Supplementary file1 (DOCX 568 kb) [file 430_2025_853_MOESM1_ESM.docx]

Supplementary material for

**Diagnostic performance of circulating microRNA signatures in differentiating tuberculosis disease from tuberculosis infection­**

Medical Microbiology and Immunology

Anne Ahrens Østergaard*^1,2^ [anne.ahrens.ostergaard@rsyd.dk](mailto:anne.ahrens.ostergaard@rsyd.dk), Stephanie Bjerrum^3,1^, Kristian Assing^4,5^, Maria Bisgaard Borup^6^, Rasmus Bank Lynggaard^7^  ,Christiane Abildgaard^1^, Ingrid Louise Titlestad^6,8^, Torben Jensen^9^, Hans Johan Niklas Lorentsson^10,11,12^, Ole Hilberg^13^, Christian Morberg Wejse^14, 15^, Søren Feddersen^7,16^, Isik Somuncu Johansen^1,2^

* Corresponding author

Affiliations:

^1^ Research Unit of Infectious Diseases, Department of Clinical Research, University of Southern Denmark, J.B. Winsløws Vej 4, indgang 20*,* 5000 Odense C, Denmark

^2^ Department of Infectious Diseases, Odense University Hospital, J.B. Winsløws Vej 4, indgang 20*,* 5000 Odense C, Denmark

^3^Department of Infectious Diseases, University Hospital of Copenhagen, Rigshospitalet, Opgang 86, 5. Sal, Esther Møllers Vej 6, 2100 København Ø, Denmark

^4^ Department of Clinical Immunology, Odense University Hospital, J.B. Winsløws Vej 4, indgang 5, 5000 Odense C, Denmark

^5^ Research unit of clinical Immunology, Department of Clinical Research, University of Southern Denmark, J.B. Winsløws Vej 4, indgang 5, 5000 Odense C, Denmark

^6^ Department of Respiratory Medicine, Odense University Hospital, J.B. Winsløws Vej 4, indgang 20, Denmark

^7^ Department of Clinical Biochemistry, Odense University Hospital, Kløvervænget 47, 5000 Odense C, Denmark

## ^8^ Odense Respiratory Research Unit (ODIN), Department of Clinical Research, University of Southern Denmark, Kløvervænget 2, Indgang 87-88, 5000 Odense C, Denmark

^9^ Department for Pulmonary Diseases, Esbjerg Hospital, Finsensgade 35, Bygning E, etage 3, 6700 Esbjerg, Denmark

^10^ Section of Infectious Diseases, Department of Medicine, Herlev and Gentofte Hospital, University of Copenhagen*,* Gentofte Hospitalsvej 1, 2900 Hellerup, Denmark

^11^Center for Clinical Metabolic Research, Herlev and Gentofte Hospital, University of Copenhagen, [Borgmester Ib Juuls Vej 83, 2730 Herlev](https://www.google.com/maps/place/data=!4m2!3m1!1s0x46524de2df139d35:0x5418c2108f17fb04?sa=X&ved=1t:8290&ictx=111), Denmark

^12^International Reference Laboratory of Mycobacteriology, Statens Serum Institut, [Artillerivej 5, 2300 København](https://www.google.com/maps/place/data=!4m2!3m1!1s0x465253411f8eb6e9:0x2f789172bc3a2b55?sa=X&ved=1t:8290&ictx=111), Denmark

^13^Department of Medicine, Vejle Hospital, Hospital Lillebælt, [Beriderbakken 4, 7100 Vejle](https://www.google.com/maps/place/data=!4m2!3m1!1s0x464c824c15ff8f85:0xd84f9e181025ec4e?sa=X&ved=1t:8290&ictx=111), Denmark

^14^ Department of Infectious Diseases, Aarhus University Hospital, [Palle Juul-Jensens Blvd. 99, 8200 Aarhus](https://www.google.com/maps/place/data=!4m2!3m1!1s0x464c158048b5ebff:0xc28c426900d506?sa=X&ved=1t:8290&ictx=111), Denmark

^15^ GloHAU, Center for Global Health, Department of Public Health, Aarhus University, Bartholins Allé 2, 8000 Aarhus C, Denmark

^16^ Clinical Biochemistry, Department of Clinical Research, University of Southern Denmark, Kløvervænget 47, 5000 Odense C, Denmark

**Diagnoses of excluded participants**

- Multiple myeloma with exacerbation of chronic obstructive lung disease, n=1
- Pulmonary *M. xenopi* infection, n=1
- Pulmonary *M. intracellulare* infection, n=1
- Alcoholic cirrhosis, n=1
- pyogenic vertebral spondylodiscitis, n=1
- Pulmonary *M. avium* infection and acquired immunodeficiency syndrome (AIDS), n=1
- Inflammatory bowel disease, n=1
- Metastatic lung cancer, n=1
- Lupus complicated by Epstein Barr Virus infection, pericarditis, nephrotic syndrome, and hemolysis, n=1

**Table S1**. **Participant** **characteristics in discovery and validation subgroups.**

| Characteristics | **Discovery group** | **Validation group** |
| --- | --- | --- |
| **Median age in years[IQR]** | | |
| - PTB | 49.2 [40.8-62.1] | 31.8 [27.2-35.2] |
| - EPTB | 43.1 [31.7-54.1] | 31.5 [27.1-41.7] |
| - Probable/possible TB | 40.8 [32.3-49.6] | 43.1 [28.3-63.3] |
| - Definite TB | 48.8 [47.8-61.7] | 30.9 [27.0-34.6] |
| - Before treatment | 48.1 [38.4-60.5] | 31.6 [27.2-39.3] |
| - After treatment | 40.8 [32.3-47.8] | 37.0 [32.2-41.2] |
| **Sex, female %** | | |
| - PTB | 30.0 | 42.9 |
| - EPTB | 75.0 | 43.8 |
| - Probable/possible TB | 66.7 | 57.1 |
| - Definite TB | 33.3 | 37.5 |
| - Before treatment | 50.0 | 43.5 |
| - After treatment | 60.0 | 50.0 |

EPTB: extrapulmonary tuberculosis, IQR: Interquartile range, PTB: pulmonary tuberculosis and TB: tuberculosis.

**Comorbidities, alcohol, smoking and substance use**:

Chronic obstructive pulmonary disease or asthma were present in four persons in the discovery group (TBI; n=4), and six persons in the validation group (TB; n=4, TBI; n=2).

Diabetes mellitus type II was present in one patient in the discovery group with EPTB and two patients within the validation group (EPTB, n=1 and PTB, n=1).

In the validation group, one participant with TBI and two with PTB were living with HIV.

Inflammatory rheumatic disease or inflammatory bowel disease were present in one person with TB from the discovery group and in seven persons in the validation group (TB; n=3, TBI; n=4).

Malignancy was present in one person with TB in the discovery group and one person with TBI in the validation group.

Hypertension was present in two with TBI in the discovery group, and seven in the validation group (TB; n=3, TBI; n=4)

In the discovery group, nine had current tobacco use (TB; n=5, TBI; n=4) and in the validation group, 20 had current tobacco use (TB; n=11, TBI; n=9).

Alcohol intake above 10 units/day was present in three from the discovery group (TB; n=2, TBI; n=1) and four from the validation group (TB; n=1, TBI; n=3).

Current substance abuse was present in two in the discovery (TB; n=2) and five in the validation group (TB; n=3, TBI; n=2)

**Figure S1:** Random forest feature importance from recursive feature elimination selected biomarkers in **A**: extrapulmonary and pulmonary tuberculosis. **B**: Tuberculosis infection vs tuberculosis **C**: Definite tuberculosis vs probable/possible tuberculosis. **D**: Tuberculosis before and after two months of anti-tuberculous treatment.


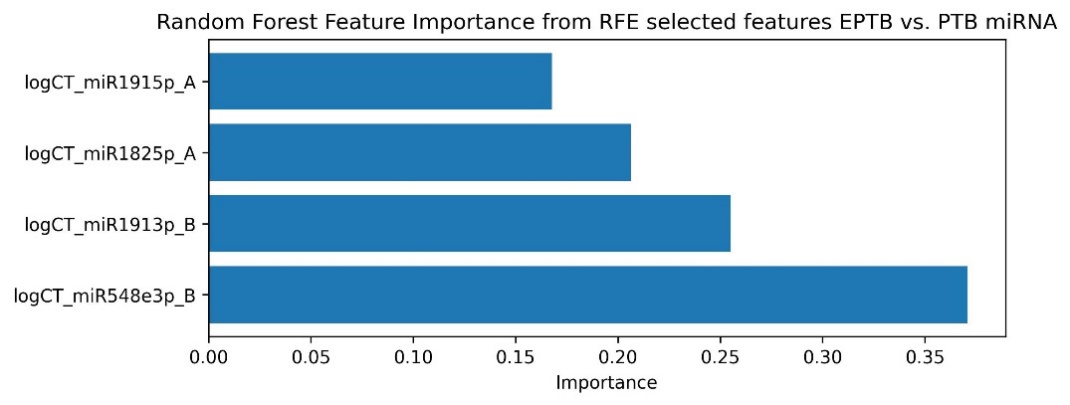
**A:**


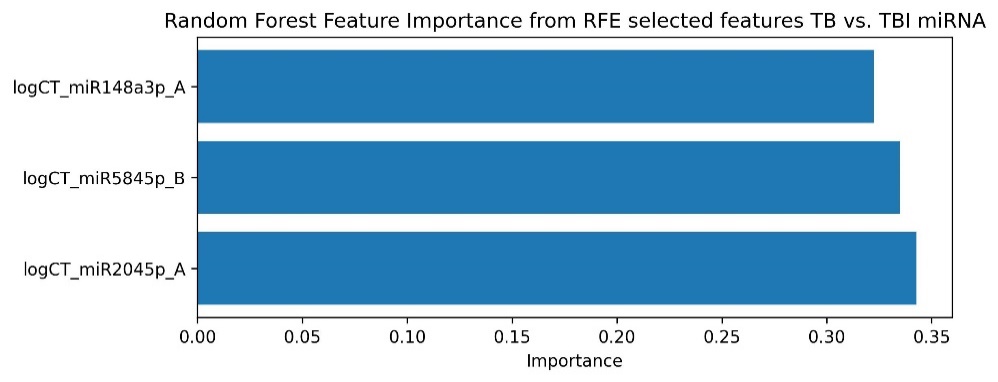
**B:**


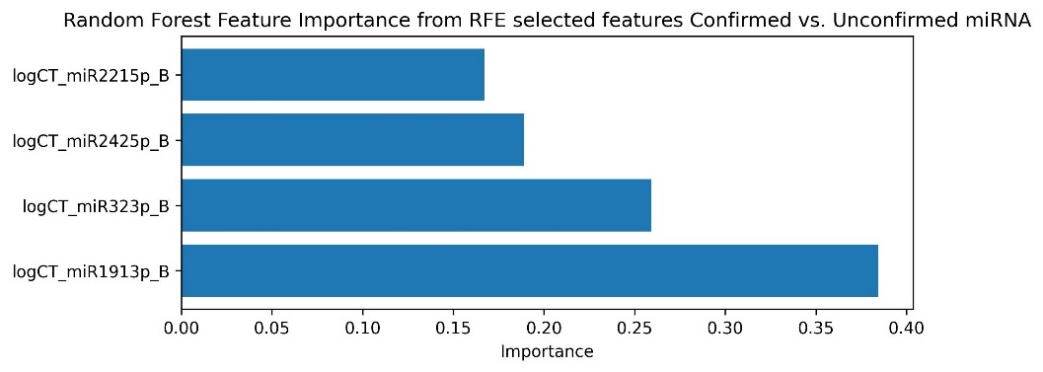
**C:**


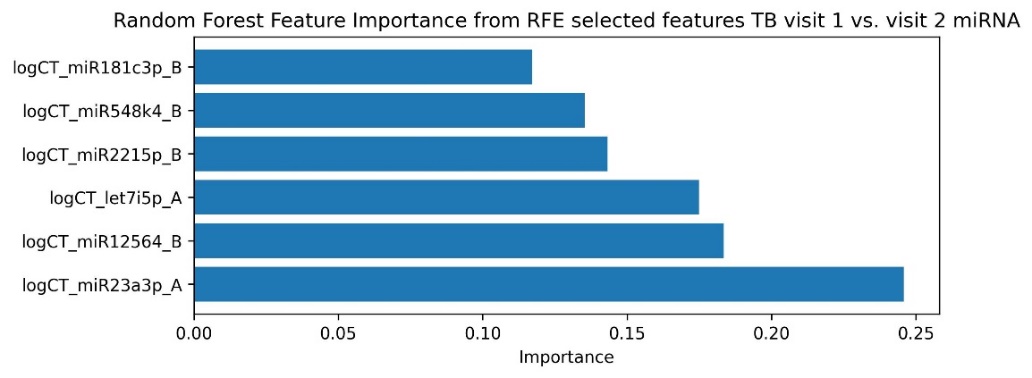
**D:**

**Figure S2.** Volcano plot of -log10 p values by log2 fold change CNRQ of 495 miRNAs in discovery group of tuberculosis and tuberculosis infection. P-values were calculated by median test. Recursive feature elimination selected miRNAs (blue) hsa-miR-148a-3p, hsa-miR-204-5p and hsa-miR-584-5p and significant miRNAs (grey) hsa-let-7a-5p, hsa-let-7e-3p, hsa-let-7f-5p, hsa-miR-1301-3p, hsa-miR-139-5p, hsa-miR-146a-5p, hsa-miR-148a-3p, hsa-miR-188-5p, hsa-miR-190a-5p, hsa-miR-204-5p, hsa-miR-22-5p, hsa-miR-33a-5p and hsa-miR-374b-5p.

**Figure S3:** Volcano plot of -log10 p values by log2 fold change spike-in normalised $\Delta$Cqs of 753 miRNAs in discovery group of tuberculosis and tuberculosis infection. P-values was calculated by median test. Recursive feature elimination selected miRNAs (blue) hsa-miR-148a-3p, hsa-miR-204-5p and hsa-miR-584-5p and significant miRNAs (grey) hsa-let-7a-5p, hsa-let-7e-3p, hsa-let-7f-5p, hsa-miR-1301-3p, hsa-miR-139-5p, hsa-miR-146a-5p, hsa-miR-148a-3p, hsa-miR-188-5p, hsa-miR-190a-5p, hsa-miR-204-5p, hsa-miR-22-5p, hsa-miR-33a-5p, hsa-miR-374b-5p and hsa-miR-584-5p.

**Figure S4.** Scatterplot of calibrated normalized relative quantities (CNRQ) values of recursive feature elimination selected miRNAs in discovery group of TB before and after 8-weeks of antituberculous treatment. P-value (p) calculated by median test for discovery group (grey) and the same miRNAs in validation group (sand) showing only significant findings. Box shows median and quartiles of both groups (black). No data in the baseline validation group for hsa-miR-15b-3p, hsa-miR-181c-3p, hsa-miR-200b-3p, hsa-miR-450a-5p and both validation groups for hsa-miR-7-1-3p.
